# Patient Delay in Colorectal Cancer Patients: Associations with Rectal Bleeding and Thoughts about Cancer

**DOI:** 10.1371/journal.pone.0069700

**Published:** 2013-07-22

**Authors:** Anette F. Pedersen, Rikke P. Hansen, Peter Vedsted

**Affiliations:** Research Unit for General Practice, The Research Centre for Cancer Diagnosis in Primary Care (CaP), Aarhus University, Aarhus C, Denmark; The University of Hong Kong, Hong Kong

## Abstract

Rectal bleeding is considered to be an alarm symptom of colorectal cancer. However, the symptom is seldom reported to the general practitioner and it is often assumed that patients assign the rectal bleeding to benign conditions. The aims of this questionnaire study were to examine whether rectal bleeding was associated with longer patient delays in colorectal cancer patients and whether rectal bleeding was associated with cancer worries. All incident colorectal cancer patients during a 1-year period in the County of Aarhus, Denmark, received a questionnaire. 136 colorectal cancer patients returned the questionnaire (response rate: 42%). Patient delay was assessed as the interval from first symptom to help-seeking and was reported by the patient. Patients with rectal bleeding (N = 81) reported longer patient intervals than patients without rectal bleeding when adjusting for confounders including other symptoms such as pain and changes in bowel habits (HR = 0.43; p = 0.004). Thoughts about cancer were not associated with the patient interval (HR = 1.05; p = 0.887), but more patients with rectal bleeding reported to have been wondering if their symptom(s) could be due to cancer than patients without rectal bleeding (chi^2^ = 15.29; p<0.001). Conclusively, rectal bleeding was associated with long patient delays in colorectal cancer patients although more patients with rectal bleeding reported to have been wondering if their symptom(s) could be due to cancer than patients without rectal bleeding. This suggests that assignment of symptoms to benign conditions is not the only explanation of long patient delays in this patient group and that barriers for timely help-seeking should be examined.

## Introduction

The 1-year relative survival of colorectal cancer is between 70–90% and has improved during the last decade. However, it continues to be lower in Denmark and UK than in other western countries [1]. One reason for these differences could be that patients in Denmark and UK wait longer for a diagnosis. The time from first symptom to first consultation is often referred to as ‘patient delay’ or the ‘patient interval’ [2], and about half of colorectal cancer patients report a patient interval of three months or longer [3], [4]. The results of a recent study revealed a U-shaped curve when examining the association between delay in cancer diagnosis and 5-year mortality in colorectal cancer patients, that is, patients with very short or very long diagnostic time intervals had higher mortality than the rest [5]. It is generally accepted that the higher mortality for patients with very short diagnostic time intervals is a result of bias inflicted by patients with fast-growing tumors who, despite immediate help-seeking, have a poor prognosis as the tumor has often spread at the time of first symptom.

Colorectal cancer can present with an array of symptoms and approximately 35–48% of patients diagnosed with colorectal cancer have experienced rectal bleeding [3], [4], [6]–[9]. Even though the positive predictive value of rectal bleeding for colorectal cancer is low (<3%) [10], it is regarded as an alarm symptom in persons over the age of 40 years [11]. Meanwhile, the majority of individuals who experience rectal bleeding do not report it to their general practitioner (GP) [12]. More surprisingly, studies have shown that colorectal cancer patients, who had experienced rectal bleeding, delayed help-seeking more often than patients who had not experienced rectal bleeding [8], [9], [13].

The possible association between rectal bleeding and patient delay differentiates colorectal cancer from most other cancers where bleeding appears to be associated with a short patient interval [14]. Therefore, it is imperative that the factors contributing to this are examined and understood. It has been assumed that the revealed association between rectal bleeding and long patient intervals is a consequence of patients attributing the rectal bleeding to benign causes such as hemorrhoids [11], [15], [16]. Meanwhile, the results of one study of 93 patients who presented with rectal bleeding to their GP suggested that the relationship between rectal bleeding and the patient interval appeared to be modified by personal experiences [11]. Thus, it was found that those patients who had experienced rectal bleeding before and may had suffered from known benign rectal disorders were *less* likely to delay help-seeking than those who had never experienced rectal bleeding before. The proportion of patients who considers cancer when experiencing rectal bleeding is not known. The results of a British population-based survey have suggested that the response to a possible cancer symptom is determined by a complex interplay between level of cancer awareness and emotional barriers. Thus, approximately 94% of the participants reported that they would contact the doctor in less than 2 weeks if they experienced an unexplained bleeding, but 37% of the same participants reported that worries about what the doctor might find would make them postpone help-seeking [17].

On this background, the aim of the present study was to examine whether patients who had experienced rectal bleeding had longer patient intervals than patients who had not experienced rectal bleeding and whether thoughts about cancer in the patient interval were associated with rectal bleeding and acted as a moderator of the relationship between rectal bleeding and the patient interval.

## Materials and Methods

### Ethics Statement

According to the Scientific Ethics Committee in the County of Aarhus, the project did not need approval by the Danish Biomedical Research Ethics Committee System. The study was approved by the Danish Data Protection Agency and the Danish National Board of Health. The data used in the study will be freely available upon request.

### Patients

The study population included all incident colon cancer (ICD-10 code: C18) and rectal cancer (ICD-10 code: 19–20) patients during a 1-year period from 1 September 2004 to 31 August 2005 in the County of Aarhus, Denmark. An incident colorectal cancer was defined as a new cancer diagnosis excluding recurrent cancers of the same type. Patients younger than 18 years were excluded. Patients were identified from the County Hospital Discharge Registry (HDR) which for each hospital admission and outpatient visit records the patient’s unique civil registration number (CRN) and diagnoses. The patient’s CRN was linked to the County Health Service Registry (HSR) to identify the patient’s GP. The patient’s GP was sent a questionnaire asking the GP to confirm the diagnosis.

Patients with a confirmed diagnosis were sent a questionnaire and non-responders received a reminder after three weeks. Besides questions concerning marital status and educational level, the questionnaire contained questions concerning the following variables:

### The Patient Interval

The patient interval was reported by the patients. Patients were asked to state the date when they first experienced a symptom that they now considered associated with their cancer disease and the date when they presented to a doctor for the first time. The patient interval was defined as between these two dates. Intervals longer than 365 days were coded as 365 days.

### Rectal Bleeding

A number of possible cancer symptoms were listed, including weight loss, pain, fatigue, changes in bowel habits, nausea/loss of appetite, general indisposition, and rectal bleeding. Patients were asked to tick off all the symptoms they had when they first considered that they could have a disease. The symptom list is provided in Questionnaire S1.

### Thoughts about Cancer

Patients were asked whether they had been wondering if their symptom(s) could be due to cancer during the patient interval. Their answer was scored on a 4-point Likert scale ranging from 0 (not at all) to 3 (very much). Patients scoring 0 were categorized as “did not have thoughts about cancer” whereas patients scoring 1 to 3 were categorized as “had had thoughts about cancer”.

### Data Analysis

Marital status was dichotomized into ‘married/cohabiting’ and ‘singles’ and educational level was dichotomized into ‘below middle-range training’ (revised International Standard Classification of Education (ISCED) level 5) and ‘middle-range training and above’ (revised ISCED level 5 to 8) [18]. The patient interval was treated as a continuous variable expected to be non-normally distributed. Associations between the patient interval and rectal bleeding and covariates were tested univariately and multivariately by hierarchical Cox time-to-event regression analysis, which can be applied to measures of event occurrence non-normally distributed [19]. The event was defined as taking contact to a doctor and time started when patients first recognised the symptom. The first model included rectal bleeding, thoughts about cancer, covariates previously suggested being associated with patient interval in colorectal cancer patients (age, gender, marital status, and educational level [14], [20]) and other symptoms reported by 20% or more of the sample. In the second model an interaction term between rectal bleeding and thoughts about cancer was added. The Kruskall-Wallis rank test was used to test differences in median length of patient intervals between groups based on the cross-classification of the categorical variables ‘rectal bleeding’ and ‘thoughts about cancer’. Data were analysed using the STATA version 11 statistical software.

## Results

During the 1-year period, a total of 327 incident colorectal cancers were identified and sent the questionnaire. Of these, 185 patients (57%) responded. Forty-nine patients (26%) did not provide the two dates necessary for calculation of the patient interval and were excluded from the analyses. Three patients reported a patient interval longer than 365 days (range: 403 days to 1295 days) and their intervals were coded as 365 days. The median patient interval of the sample (n = 136; response rate = 42%) was 28 days (interquartile interval 5 to 70 days). Characteristics of the sample are presented in Table 1.

**Table 1:** Characteristics of the patient sample.

	All N = 136	Rectal bleeding N = 81 (59.56%)	No rectal bleedingN = 55 (40.44%)	Test of difference for rectal bleeding or not
Age, mean (sd)	67.88 (11.86)	66.48 (12.36)	69.93 (12.36)	t = 1.67, p = 0.097
Females, N (%)	61 (44.85)	35 (43.21)	26 (42.27)	chi^2^ = 0.23, p = 0.640
Marital status				
Married/cohabiting, N (%)	93 (68.38)	63 (77.78)	30 (54.55)	
Single, N (%)	40 (29.41)	17 (20.99)	23 (41.82)	chi^2^ = 7.43, p = 0.006
Missing information, N (%)	3 (2.21)	1 (1.23)	2 (3.64)	
Educational level				
< Middle-range training, N (%)	87 (63.97)	50 (61.73)	37 (67.27)	
≥ Middle-range training, N (%)	44 (32.35)	30 (37.04)	14 (25.45)	chi^2^ = 4.76, p = 0.092
Missing information, N (%)	5 (3.68)	1 (1.23)	4 (7.27)	
Diagnosis				
Rectal cancer, N (%)	54 (39.71)	41 (50.62)	13 (23.64)	
Colon cancer, N (%)	82 (60.29)	40 (49.38)	42 (76.36)	chi^2^ = 9.96, p = 0.002
Had thoughts about cancer				
No, N(%)	65 (47.79)	30 (37.04)	35 (63.64)	
Yes, N (%)	66 (48.53)	50 (61.73)	16 (29.09)	chi^2^ = 15.29, p<0.001
Missing information, N (%)	5 (3.68)	1 (1.23)	4 (7.27)	
Patient delay, median (IQI)	28 (5–70)	39 (9–96)	15 (2–31)	z = 3.41, p<0.001

IQI = Interquartile interval.

None of the patients were asymptomatic when they sought medical help, and a total of 81 patients (60%) had experienced rectal bleeding during the patient interval. Additional to rectal bleeding, the most commonly reported symptoms, i.e. symptoms reported by 20% or more of the sample, were changes in bowel habits (65%), fatigue (47%), pain (35%), weight loss (21%), and general indisposition (20%). Among the rarely reported symptoms were dizziness (13.2%), lack of appetite/nausea (11.8%) and fever (5.2%). A total of 14 (10%) patients had experienced rectal bleeding without co-occurrence of any of the other five commonly reported symptoms. As revealed in Table 1, patients who had experienced rectal bleeding reported a statistically significant longer median patient interval of 39 days compared to 15 days in patients who had not experienced rectal bleeding. In Table 2, the median patient intervals in days are reported for patients, who reported changes in bowel habits, fatigue, pain, weight loss, and general indisposition either in combination with rectal bleeding or not in combination with this symptom.

**Table 2:** Median patient interval (in days) for the five symptoms occurring in ≥20% of the sample (N = 136).

	Changes inbowel habits	Pain	Weight loss	Fatigue	Generalindisposition
**Median (IQI) patient interval when** **presented without rectal bleeding**	16 (5–31)	14 (3–28)	18 (4–29)	17 (4–29)	10 (0–29)
	N = 30 (22.1%)	N = 25 (18.4%)	N = 17 (12.5%)	N = 26 (19.1%)	N = 11 (8.1%)
**Median (IQI) patient interval when** **presented together with rectal bleeding**	61 (12–112)	31 (13–119)	38 (22–74)	34 (5–96)	31 (0–57)
	N = 58 (42.6%)	N = 22 (16.2%)	N = 12 (8.8%)	N = 38 (27.9%)	N = 16 (11.8%)

Median patient interval in the 14 patients with rectal bleeding and none of the five common symptoms was 22 days (IQI = 3–42 days).

IQI = Interquartile interval.

The Cox regression models revealed that rectal bleeding was associated with longer patient intervals when adjusting for the influence of covariates, including the other five most commonly reported symptoms (Table 3). None of the other covariates, including thoughts about cancer, were associated with length of patient interval.

**Table 3:** Summary of hierarchical Cox regression analysis with length of the patient delay as dependent variable (N = 136).

	Univariate analyses			Multivariate analysis, Model 1			Multivariate analysis, Model 2		
	HR	95% CI	P-value	HR^a^	95% CI	P-value	HR^a^	95% CI	P-value
Rectal bleeding									
No	1.00			1.00			1.00		
Yes	0.48	0.33–0.69	0.000	0.48	0.31–0.76	0.002	0.43	0.25–0.77	0.004
Thoughts about cancer									
No	1.00			1.00			1.00		
Yes	0.97	0.69–1.37	0.870	1.26	0.83–1.92	0.278	1.05	0.51–2.16	0.887
Age	1.01	0.99–1.02	0.382	1.00	0.99–1.02	0.748	1.00	0.98–1.02	0.829
Gender									
Female	1.00			1.00			1.00		
Male	0.87	0.62–1.23	0.441	0.83	0.55–1.25	0.366	0.83	0.55–1.26	0.379
Diagnosis									
Rectal cancer	1.00			1.00			1.00		
Colon cancer	1.25	0.89–1.77	0.199	0.91	0.54–1.51	0.709	0.92	0.55–1.54	0.478
Marital status									
Single	1.00			1.00			1.00		
Married/cohabiting	0.75	0.52–1.09	0.134	0.84	0.54–1.29	0.421	0.83	0.54–1.28	0.403
Educational level									
< Middle-range training	1.00			1.00			1.00		
≥ Middle-range training	0.84	0.58–1.21	0.338	0.97	0.65–1.44	0.887	0.98	0.66–1.46	0.937
Rectal bleeding x Thoughts about cancer							1.32	0.54–3.27	0.543

HR = Hazard ratio; CI = Confidence Intervals; HR^a^ : adjusted for the presence of other symptoms including changes in bowel habits, pain, weight loss, fatigue, and general indisposition.

**Figure 1:**
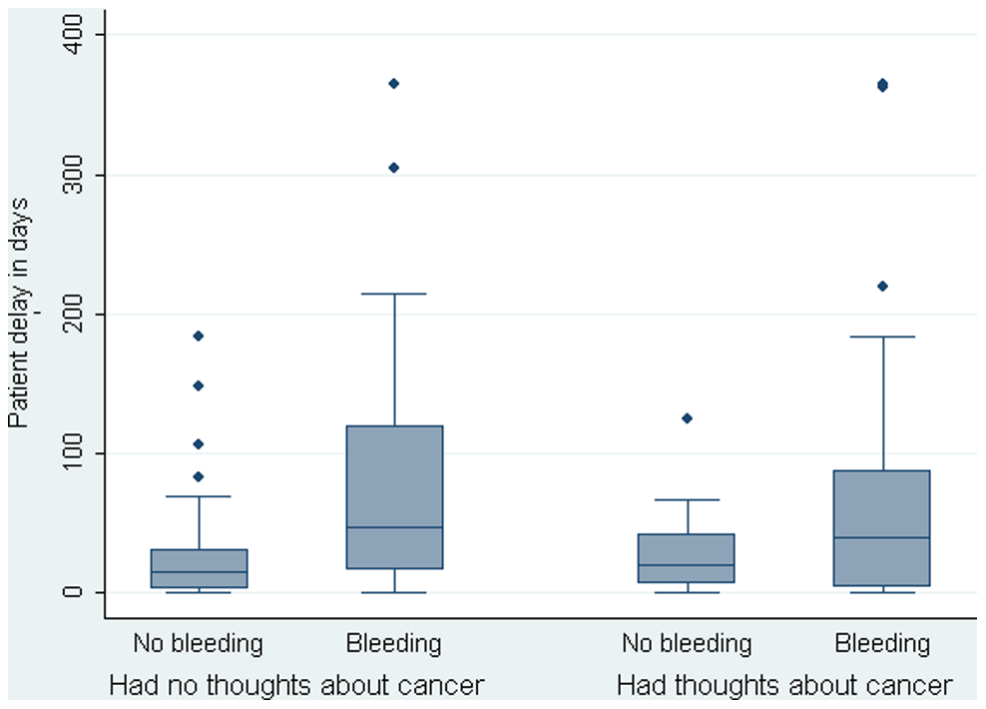
The association between rectal bleeding and length of the patient delay in patients who had thoughts about cancer and in patients who did not have thoughts about cancer.

As shown in Table 1, both length of the patient interval and thoughts about cancer were related to the experience of rectal bleeding. The median patient intervals and the interquartile intervals (IQI) in the four groups developed by cross-classifying the two categorical variables ‘rectal bleeding’ and ‘thoughts about cancer’ are shown in Figure 1. The Kruskall-Wallis test documented a significant difference in median patient interval between the groups (Chi^2^ = 10.80, p = 0.01). As shown, patients who had experienced rectal bleeding and had no thoughts about cancer, reported the longest patient intervals (median = 46 days; IQI = 16–119 days) whereas patients who had not experienced rectal bleeding and had no thoughts about cancer reported the shortest patient intervals (median = 15 days; IQI = 2–31 days). The median patient interval in patients who had experienced rectal bleeding and had thoughts about cancer was 39 days (IQI = 3–87 days) and 19 days (IQI = 6–41 days) in patients who had not experienced rectal bleeding, but had thoughts about cancer. The median patient intervals in the groups did not suggest that the influence of rectal bleeding on length of patient interval was dependent on whether the patients reported thoughts about cancer and formal testing did no either document such an interaction effect (see Table 3).

## Discussion

Patients with rectal bleeding reported longer patient intervals than patients without rectal bleeding. The difference between the groups was clear with patients who had experienced rectal bleeding reporting a patient interval of 39 days and 15 days in patients who had not experienced rectal bleeding. Thoughts about cancer were not associated with the patient interval and did not act as a moderator on the relationship between rectal bleeding and long patient intervals, that is the association between rectal bleeding and longer patient intervals was not dependent on whether the patients reported to have had thoughts about cancer in the period from first symptom to medical help seeking. However, more patients with rectal bleeding reported to have been wondering if their symptom(s) could be due to cancer during the patient interval than patients without rectal bleeding.

A reasonably high number of participants and the use of a reliable Danish register for identification of patients are among the strengths of this study. The use of a reliable register secured that all incident colon cancer and rectal cancer patients were invited to participate. However, a number of limitations of the present study should also be noted. First, the relatively low participation rate on 42% may have influenced the generalizability of our results. The proportion of returned questionnaires was higher (57%) and similar to what has been reported before (see for instance [4]), but unfortunately, many patients had difficulty reporting the dates necessary for calculation of the patient interval and had to be excluded. This could reflect the downside of identifying patients through a register as it made any prior evaluation of the course preceding the cancer diagnosis impossible. Insofar patients were recruited on hospital wards and data were obtained through interviews, patients could be helped to determine the dates. Second, the cross-sectional study design does not allow determination of the direction of causality. Third, the study is retrospective and we cannot exclude possible recall bias, a well-known methodological limitation in studies of patient interval [21]. The recall may give rise to an information bias in relation to the time of the first experienced symptom because an alarm symptom such as rectal bleeding may be easier to remember than other unspecific symptoms. Despite that it is difficult to determine whether this possible bias has influenced the results, we are inclined to believe that it would underestimate the patient interval. Thus, an alarm symptom might overshadow an eventual unspecific symptom experienced previously, e.g. people may forget changing bowel habits if they later on experience rectal bleeding. This would give an impression of a shorter patient interval for alarm symptoms, thereby tending to underestimate the association found in our study. Fourth, the patient’s history of prior anorectal diagnoses such as hemorrhoids would have been of interest, but unfortunately this information had not been recorded. We strongly recommend that future studies assess patients’ history of prior anorectal diagnoses. The prevalence of symptomatic hemorrhoids in the adult population is 20% [22] and, to our knowledge, there is no evidence to support that the prevalence of hemorrhoids in colorectal cancer patients should be different compared to the level observed in the general population or unevenly distributed among patients with or without rectal bleeding. As only one fifth of the patients in the study would have hemorrhoids, at least according to statistical figures supposedly applicable to patients both with and without rectal bleeding, the influence would be relatively small. Insofar we assume that the long delay in the group of patients with rectal bleeding can be explained by a past history of hemorrhoids, one should expect that patients with rectal bleeding worried less about cancer in the patient interval than patients without rectal bleeding. Meanwhile, we found the opposite result, namely that patients with rectal bleeding reported to have thought about cancer more than patients without rectal bleeding.

The association between rectal bleeding and longer patient intervals has also been documented in previous research [8], [9], [13]. The association between rectal bleeding and more thoughts about cancer appears to contradict the assumption that a long patient interval in patients with rectal bleeding should be caused solely by assigning the symptom to benign causes. The results of the present study may suggest that emotional barriers such as embarrassment about symptoms and fear of diagnostic procedures should be taken into consideration when addressing interventions aimed at promoting timely help-seeking in patients with any possible cancer symptom [20], [23].

In the general population, worries about what the doctor might find have been shown to be a prominent barrier for help-seeking [17]. This may explain our somewhat counterintuitive finding that patients without rectal bleeding (i.e. an alarm symptom) and without thoughts about cancer (i.e. no cancer worries) had the shortest patient interval. When examining the influence of thoughts about cancer on the patient interval, wide confidence intervals were, however, revealed. This suggests that the influence of cancer worries on the patient interval is not uniform within the patient group. From research conducted among breast cancer patients, it has been documented that recognition of symptom seriousness can inflict both short and long patient intervals depending on the patients’ coping response, i.e. whether the patient uses avoidant or confronting coping strategies [14], [24]. Whether an unexplained symptom elicit an avoidant or confronting coping response may depend on whether the individual perceives himself to be able to handle the anticipated health threat [25]. If fear about having a serious disease may paradoxically prolong the patient interval in some patients, it will be important that health campaigns raise awareness of symptoms and signs of serious diseases in a way which challenge an exaggerated pessimistic attitude [26].

In questionnaire studies of the general population, 14–33% report that they have experienced rectal bleeding at some time in their life, and rectal bleeding in the last year is reported by 6–19% depending on the age of the responders [27], [28]. In the general population, the positive predictive value of rectal bleeding is estimated to be 0.1%, but once the symptom has been reported to the GP, the positive predictive value rises to approximately 3% [28]. This suggests that patients can identify which symptoms matter, but the long patient intervals documented in colorectal cancer patients underline the need for better decision aids for patients facing a symptom which is probably a symptom of a benign condition, but can be a sign of cancer.

In conclusion, the results of the present study revealed that patients who had experienced rectal bleeding reported longer patient intervals compared to patients who had not experienced rectal bleeding when controlling for the influence of possible confounders and other commonly reported symptoms. The results did not document that the observed association between rectal bleeding and long patient intervals was moderated by whether the patients had been wondering if their symptom(s) could be due to cancer, but patients with rectal bleeding were more inclined to have thought about cancer than patients without rectal bleeding, and this finding seems to question whether long patient intervals in patients experiencing rectal bleeding are solely a consequence of assigning the symptom to benign causes.

## Supporting Information

Questionnaire S1 The questions included in this study.
(DOC)
