# Supplementary material for: Patient Delay in Colorectal Cancer Patients: Associations with Rectal Bleeding and Thoughts about Cancer
Source: PLoS One. 2013 Jul 22;8(7):e69700. doi: 10.1371/journal.pone.0069700 (PMC3718764; doi:10.1371/journal.pone.0069700)
Supplement: Questionnaire S1 — The questions included in this study. (DOC) [file pone.0069700.s001.doc]

**Questionnaire S1.**

*The questions included in this study*

At which (approximate) date did you notice, for the first time, signs or symptoms that could be caused by your cancer disease?

At which date did you attend a consultation with your GP or receive a house call for the first time because of cancer suspicion?

How did you react when you suspected for the first time that something could be wrong with your health?

I thought it could be cancer

Response categories: Not at all; To some extent; A great deal; To a great extent

Which symptoms and possible signs of disease did you have when you suspected for the first time that something could be wrong with your health? (you may select several categories)

Lump (incl. swollen lymph nodes)

Weight loss

Pain

Changes in bowel habits

Rectal bleeding/blood in stools

Fatigue

Fever

Dizziness

Loss of appetite/nausea

Increased perspiration

General indisposition

Other things, please describe:

(The data collection was carried out as part of a survey including patients with other types of cancer. The symptoms listed above are the symptoms relevant for colon cancer and rectal cancer).
